# Nasopharyngeal carcinoma

**DOI:** 10.1186/1750-1172-1-23

**Published:** 2006-06-26

**Authors:** Bernadette Brennan

**Affiliations:** 1 Royal Manchester Children's Hospital, Hospital Road, M27 4HA Manchester, UK

## Abstract

Nasopharyngeal carcinoma (NPC) is a tumor arising from the epithelial cells that cover the surface and line the nasopharynx. The annual incidence of NPC in the UK is 0.3 per million at age 0–14 years, and 1 to 2 per million at age 15–19 years. Incidence is higher in the Chinese and Tunisian populations. Although rare, NPC accounts for about one third of childhood nasopharyngeal neoplasms. Three subtypes of NPC are recognized in the World Health Organization (WHO) classification: 1) squamous cell carcinoma, typically found in the older adult population; 2) non-keratinizing carcinoma; 3) undifferentiated carcinoma. The tumor can extend within or out of the nasopharynx to the other lateral wall and/or posterosuperiorly to the base of the skull or the palate, nasal cavity or oropharynx. It then typically metastases to cervical lymph nodes. Cervical lymphadenopathy is the initial presentation in many patients, and the diagnosis of NPC is often made by lymph node biopsy. Symptoms related to the primary tumor include trismus, pain, otitis media, nasal regurgitation due to paresis of the soft palate, hearing loss and cranial nerve palsies. Larger growths may produce nasal obstruction or bleeding and a "nasal twang". Etiological factors include Epstein-Barr virus (EBV), genetic susceptibility and consumption of food with possible carcinogens – volatile nitrosamines. The recommended treatment schedule consists of three courses of neoadjuvant chemotherapy, irradiation, and adjuvant interferon (IFN)-beta therapy.

## Definition

Nasopharyngeal carcinoma (NPC) is a tumor arising from the epithelial cells that cover the surface and line the nasopharynx. NPC was first described as a separate entity by Regaud and Schmincke in 1921 [1,2]. Approximately one third of nasopharyngeal carcinomas of the undifferentiated type are diagnosed in adolescents or young adults. Although rare, NPC accounts for one third of childhood nasopharyngeal neoplasms (data from USA) [3].

## Epidemiology

The annual incidence of NPC in the UK is 0.25 per million (age standardized, age 0–14 years), 0.1 per million at age 0–9 years and 0.8 per million at age 10–14 years. It seems reasonable to assume, on the basis of England and Wales cancer registry data, that at least 80% of nasopharyngeal cancers at age 15–19 years are carcinomas. This suggests an incidence of 1 to 2 per million for NPC at age 15–19 years.

In comparison with other countries, the incidence in the UK is low. In particular, in Tunisia the incidence is relatively high [4]. In southern parts of China, Southeast Asia, the Mediterranean basin and Alaska the incidence of NPC is moderately elevated; an incidence of 2 per million of NPC in China has been reported [5]. In other countries, for example in India, the incidence is comparable to that in the UK at 0.9 per million. Furthermore, the younger age peak in the second decade found in India [6], is also found in the UK [7].

## Etiology

NPC is the commonest epithelial cancer in adults. The detection of nuclear antigen associated with Epstein-Barr virus (EBNA) and viral DNA in NPC type 2 and 3, has revealed that EBV can infect epithelial cells and is associated with their transformation [8]. The etiology of NPC (particularly the endemic form) seems to follow a multi-step process, in which EBV, ethnic background, and environmental carcinogens all seem to play an important role.

Lo *et al*. showed that EBV DNA was detectable in the plasma samples of 96% of patients with non-keratinizing NPC, compared with only 7% in controls [9]. More importantly, EBV DNA levels appear to correlate with treatment response [9-11] and they may predict disease recurrence [11], suggesting that they may be an independent indicator of prognosis [12].

In adults, other likely etiological factors include genetic susceptibility, consumption of food (in particular salted fish) containing carcinogenic volatile nitrosamines, and as in children, EBV [13,15-19].

## Clinical presentation

NPC usually originates in the lateral wall of the nasopharynx, which includes the fossa of Rosenmuller. It can then extend within or out of the nasopharynx to the other lateral wall and/or posterosuperiorly to the base of the skull or the palate, nasal cavity or oropharynx. It then typically metastases to cervical lymph nodes. Distant metastases may occur in bone, lung, mediastinum and, more rarely, the liver.

Cervical lymphadenopathy is the initial presentation in many patients, and the diagnosis of NPC is often made by lymph node biopsy. Symptoms related to the primary tumor include trismus, pain, otitis media, nasal regurgitation due to paresis of the soft palate, hearing loss and cranial nerve palsies. Larger growths may produce nasal obstruction or bleeding and a "nasal twang". Metastatic spread may result in bone pain or organ dysfunction. Rarely, a paraneoplastic syndrome of osteoarthropathy may occur with widespread disease.

## Histopathology

Three subtypes of NPC are recognized in the World Health Organisation (WHO) classification [20]:

• type 1: squamous cell carcinoma, typically found in the older adult population

• type 2: non-keratinizing carcinoma

• type 3: undifferentiated carcinoma

Most cases in childhood and adolescence are type 3, with a few type 2 cases [21]. Type 2 and 3 are associated with elevated Epstein-Barr virus titers, but type 1 is not [22]. The Cologne modification of the WHO scheme by Krueger and Wustrow [23] includes the degree of lymphoid infiltration. Types 2 and 3 may be accompanied by an inflammatory infiltrate of lymphocytes, plasma cells, and eosinophils, which are abundant, giving rise to the term lymphoepithelioma. Two histological patterns may occur: Regaud type, with a well-defined collection of epithelial cells surrounded by lymphocytes and connective tissue, and Schmincke type, in which the tumor cells are distributed diffusely and intermingle with the inflammatory cells. Both patterns may be present in the same tumor.

## Diagnostic methods

Diagnostic methods include:

1. Clinical evaluation of the size and location of cervical lymph nodes.

2. Indirect nasopharyngoscopy to assess the primary tumor.

3. Neurological examination of cranial nerves.

4. Computed tomography (CT)/magnetic resonance imaging (MRI) scan of the head and neck to below clavicles to assess base of skull erosion.

5. Chest radiotherapy (anteroposterior and lateral) to see if NPC has spread to the lungs.

6. Bone scintigraphy by Tc 99 diphosphonate to show whether cancer has spread to the bones.

7. Full blood count.

8. Urea, electrolyte, creatinine, liver function, Ca, PO_4_, alkaline phosphate.

9. EBV viral capsid antigen and EBV DNA.

10. Biopsy of either the lymph nodes or primary tumor for histological examination.

## Staging

The tumor, node, metastasis (TNM) classification of the American Joint Committee on Cancer [24] – sixth edition (Table 1) is usually used to determine the tumor staging (Table 2). This latest TNM classification takes into account Ho's [25] modifications for NPC, which utilizes the prognostic importance of affected nodes extending into the lower cervical and supraclavicular areas.

**Table 1:** The tumor, node, metastasis (TNM) classification of the American Joint Committee on Cancer [24]

***Primary Tumor (T)***	
**TX**	Primary tumor cannot be assessed
**TO**	No evidence of primary tumor
**Tis**	Carcinoma *in situ*

***Nasopharynx***	
**T1**	Tumor confined to the nasopharynx
**T2**	Tumor extends to soft tissues of oropharynx and/or nasal fossa
• T2a	• without parapharyngeal extension
• T2b	• with parapharyngeal extension
**T3**	Tumor invades bony structures and/or paranasal sinuses
**T4**	Tumor with intracranial extension and/or involvement of cranial nerves, infratemporal fossa, hypopharynx, or orbit, or masticator space

***Regional Lymph Nodes (N): Nasopharynx***	
The distribution of regional lymph node spread from nasopharyngeal cancer, particularly of the undifferentiated type, is different than that of other head and neck mucosal cancers and justifies use of a different N classification scheme. In children this does not have a prognostic impact.		
**NX**	Regional lymph nodes cannot be assessed
**N0**	No regional lymph node metastasis
**N1**	Unilateral metastasis in lymph node(s), 6 cm or less in greatest dimension, above the supraclavicular fossa.
**N2**	Bilateral metastasis in lymph node (s) 6 cm or less in greatest dimension, above the supraclavicular fossa
**N3**	Metastasis in a lymph node(s)
• **N3a**	• greater than 6 cm in dimension
• **N3b**	• extension to the supraclavicular fossa

***Distant Metastasis (M)***	
**MX**	Distant metastasis cannot be assessed
**M0**	No distant metastasis
**M1**	Distant metastasis

**Table 2:** Stage grouping

**Stage 0**	**Tis**	**N0**	**M0**
Stage 1	T1	N0	M0
Stage IIA	T2a	N0	M0
Stage IIB	T1	N1	M0
	T2	N1	M0
	T2a	N1	M0
	T2b	N1	M0
Stage III	T1	N2	M0
	T2a	N2	M0
	T2b	N2	M0
	T3	N0	M0
Stage IVA	T4	N0	M0
	T4	N1	M0
	T4	N2	M0
Stage IVB	Any T	N3	M0
Stage IVC	Any T	Any N	M1

## Treatment

### Surgery

Due to the anatomical position of NPC and its tendency to present with cervical lymph node metastases, it is not amenable to surgery for local control. Biopsy of the involved lymph node is the usual surgical procedure. The nasopharyngeal primary tumor is rarely biopsied.

### Chemotherapy

Several factors are taken into account in deciding the chemotherapy regimen.

Firstly, efficacy: the figures for event-free survival are similar for most small chemotherapy series but therapy usually involves fairly high-dose radiotherapy to the nasopharynx – 60 to 65 Gy. However, the most promising results with a recent update, are those obtained using the Mertens protocol NPC-91-GPOH (Society of Pediatric Oncology and Hematology). This protocol should therefore be considered as the best current treatment. Uniquely, the NPC-91-GPOH protocol includes immunotherapy with interferon-beta after chemotherapy and radiotherapy, which may explain its superior results compared to regimens without interferon treatment [27].

Secondly, late effects: in terms of chemotherapy, the Manchester regimen – doxorubicin, methotrexate and cyclophosphamide – would produce infertility in boys (total dose of cyclophosphamide 12 gm/m^2^) and possible anthracycline toxicity (total dose of doxorubicin 360 mg/m^2^) [36]. The NPC-91-GPOH protocol might produce some infertility in older boys but the total dose of cisplatinum is only 300 mg/m^2^. Furthermore, the incidence of renal toxicity should be relatively low but auditory toxicity would be higher because of the additional effect of irradiation on the auditory apparatus. The degree of pituitary dysfunction obviously depends on the radiotherapy field and, potentially, on the dose of radiotherapy but some degree of hypopituitarism is expected. Furthermore, irradiation to the neck would result in hypothyroidism for the majority of patients and irradiation to the oropharynx would result in xerostomia and resultant poor dentition. The later may be relieved by amifostine, as demonstrated in adult studies.

### Radiotherapy

Although treatment with radiotherapy controls the primary tumor [28-30], it does not prevent the appearance of distant metastases [28,31].

Radiotherapy is given with megavoltage equipment after initial chemotherapy. A maximum dose of 45 Gy is given to the clinical target volume, which is a 1 cm margin around the MRI-detected primary site, and inferiorly down to the clavicles to include the lymph nodes. Treatment is given in two phases:

• Phase I – parallel pair (mostly lateral unless the tumor extends anteriorly between the eyes). Eyes, brain and brain stem are shielded as much as possible. A mid-plane dose of 30 Gy in 15 fractions is given.

• Phase II – a lateral parallel pair or three-fields technique is used for the primary site, delivering 15 Gy in seven fractions to the clinical target volume of the tumor with a 1 cm margin. Brain stem and eyes should be shielded. Any overlap with the neck field should be shielded. A matching anterior neck node field is used to deliver a prescribed maximum subcutaneous dose of 15 Gy in seven fractions. The spinal cord should be shielded in this field. This prescription for radiotherapy is used in Manchester, but it is recognized that higher doses may be used in some centers, possibly to a total of 60 Gy to the tumor volume. In an current GPOH study, patients in complete remission (CR) after three courses of chemotherapy, will have their radiotherapy dosage reduced to 54 Gy instead of 59 Gy.

### Recommendation

In the current GPOH protocol NPC-2003-GPOH, low-risk patients with Stage I and II tumors receive radiotherapy only, followed by 105 **μ**g/Kg of adjuvant interferon beta (IFNbeta), intravenously (i.v.), three times a week for 6 months. High-risk patients receive cisplatinum (100 mg/m^2 ^over 6 hours on day 1 with standard hydration), mannitol and electrolyte replacement, and folinic acid (25 mg/m^2 ^every 6 hours for a total of six doses) as well as 5-fluorouracil (1000 mg/m^2 ^per day from day 2 for 5 days) as a continuous infusion. They receive three courses of chemotherapy every 21 days or on full blood count recovery, followed by irradiation and IFNbeta as for low-risk patients. Methotrexate has been dropped because of severe mucositis. Patients not in CR after three courses of chemotherapy will receive concomitant cisplatinum (20 mg/m^2^/day for 3 days with radiotherapy for two courses).

## Prognostic factors

Presentation with lymphadenomegalia implies that the disease has spread beyond the primary site. However, in childhood the presence of metastatic disease in cervical lymph nodes at diagnosis does not adversely affect prognosis [30-33]. Factors associated with a poor prognosis are skull base involvement [33-35], extent of the primary tumor [31,32] and cranial nerve involvement [33,34].

## Unresolved questions

1. What is the optimum radiotherapy dose?

2. Would exclusion of interferon from the treatment produce similar results?

3. The relationship between late effects and dose of radiotherapy should be investigated, as well as the exact nature and incidence of late effects.
